# Gut Microbiome Alterations Accompany Metabolic Normalization Following Bariatric Surgery in ROHHAD Syndrome

**DOI:** 10.1210/jcemcr/luae091

**Published:** 2024-06-03

**Authors:** Alessandra Granato, Paul M Ryan, Anthony Wong, Jill K Hamilton, Jayne S Danska

**Affiliations:** Genetics and Genome Biology, The Hospital for Sick Children, Toronto, ON, M5G 1H3, Canada; Department of Paediatrics, University of Toronto, Toronto, ON, M5G 1X8, Canada; Genetics and Genome Biology, The Hospital for Sick Children, Toronto, ON, M5G 1H3, Canada; Department of Paediatrics, University of Toronto, Toronto, ON, M5G 1X8, Canada; Division of Endocrinology, The Hospital for Sick Children, Toronto, ON, M5G 1X8, Canada; Genetics and Genome Biology, The Hospital for Sick Children, Toronto, ON, M5G 1H3, Canada; Department of Immunology, University of Toronto, Faculty of Medicine, Toronto, ON, M5G 1X8, Canada; Department of Medicine Biophysics, University of Toronto, Faculty of Medicine, Toronto, ON, M5G 1L7, Canada

**Keywords:** obesity, ROHHAD, microbiome, bariatric surgery, gastroenterology

## Abstract

Rapid onset obesity with hypoventilation, hypothalamic, and autonomic dysregulation (ROHHAD) syndrome in childhood is characterized by abrupt onset weight gain and dysautonomia with variable neuroendocrine involvement. In the absence of definitive disease-modifying therapies, the primary management strategy remains symptom control. This case report describes the first successful correction of obesity, dysautonomia, and metabolic derangement in a patient with ROHHAD following Roux-en-Y gastric bypass. Anthropometrics, metabolic profiling, and stool microbiome composition were assessed in a longitudinal fashion. In the 48-month period following surgery, the patient body mass index (BMI) reduced by 9.5 kg/m^2^ and metabolic status improved, evidenced in weaning of insulin, and improved glycated hemoglobin, lipid profile, and hepatic enzymes. Chronic diarrhea resolved after surgery and prior to significant weight loss. Evaluation of stool bacterial composition and biomass demonstrated shifts in absolute abundance and taxonomic composition in longitudinal samples following surgery. This case demonstrates the potential efficacy of bariatric surgery in correcting the metabolic disruption of ROHHAD syndrome, producing long-term changes in gut microbiome composition and biomass.

## Introduction

Rapid onset obesity with hypothalamic dysregulation, central hypoventilation, and autonomic dysregulation (ROHHAD) is a rare syndrome with significant morbidity and mortality. A recent meta-analysis of all available published cases (*n* = 43) documents the disease course of the condition in childhood (1). Typically, rapid obesity onset in early childhood is followed by diagnosis of dysautonomia, pituitary hormone deficiencies, and hypoventilation. Neural crest/neuroendocrine tumors have occurred in approximately half of the reported cases. ROHHAD etiology remains unclear, and diagnosis is made based on clinical presentation, after ruling out genetic causes of central hypoventilation syndrome. Without definitive treatments, supportive therapies to address symptoms are employed. Here, we report the longitudinal clinical course and gut microbiome modulation of a ROHHAD patient during successful therapy, inclusive of bariatric surgery.

## Case Presentation

The patient was referred at age 12 ½ years for unexplained weight gain from the age of 4 years. At referral, their BMI was 39.2 kg/m^2^ (> 99.9%; BMIz +3.41), with a weight of 97.3 kg (> 99.9%) and height of 157.5 cm (69.3%). Her parents, sister, and twin brother had BMIs within the normal range, and a history of unexplained rapid weight gain was lacking within the family. The patient reported symptoms of autonomic dysfunction: namely diaphoresis, flushing, and frequent watery bowel movements associated with abdominal cramping and frequent day and night-time encopresis.

## Diagnostic Assessment

Multiple tests performed to evaluate Cushing syndrome demonstrated inconsistent cortisol elevation. The patient had one elevated 24-hour urinary free cortisol of 234 nmol/d or 85 µg/d (normal range: 0-151 nmol/d, 0-55 µg/d) at 12 ¾ years, followed by 2 normal values of 45 nmol/d or 16 µg/d (normal range 0-193, 0-70 µg/d) and 59 nmol/d or 21 µg/d (normal range: 0-151, 0-55 µg/d) the following year. Morning cortisol following a 1 mg dexamethasone suppression test at 13 ½ years was partially suppressed (110 nmol/L or 40 µg/L; normal range: 0-80 nmol/L, 0-29 µg/L). A repeat test 1 year later revealed cortisol suppression (< 50 nmol/L). From 12 and 14 years, the patient completed 5 midnight salivary cortisol tests with results of 0.4 to 1.1 (normal range < 1.9 nmol/L). Although cyclic Cushing syndrome was considered given the intermittent and mild nature of her hypercortisolism, this diagnosis was not deemed explanatory in absence of clinical stigmata (ie, hypertension, cushingoid facies, striae). Thyroid testing was normal (thyrotropin 2.4 mIU/L; normal 0.4-4.2 mIU/L).

Metabolic screening identified type 2 diabetes, with glycated hemoglobin (HbA1c) ranging from 9.5% to 11.9% (80-107 mmol/mol; normal range: < 6.5% or < 48 mmol/mol) and hypertriglyceridemia (26-85 mmol/L or 2303-7529 mg/dL; normal range: < 1.7 mmol/L or < 150 mg/dL). The patient developed secondary amenorrhea and hirsutism suggestive of polycystic ovarian syndrome, as well as metabolic dysfunction-associated fatty liver disease with transaminitis (alanine aminotransferase [ALT] 43-68 IU/L; normal range: < 40 IU/L) and hepatopancreatic fatty infiltrations.

Sleep assessment identified obstructive sleep apnea and central hypoventilation, with persistent hypercarbia > 50 mmHg (0.66 kPa; normal range: 35-45 mmHg or 0.47-0.6 kPa) and regular desaturations to 50% SpO2. Genetic testing for *PHOXB* mutation-associated central hypoventilation was negative and brain imaging was normal. Biphasic positive airway pressure at night was effective.

To explore potential causes of her persistent dysautonomia, upper and lower endoscopy, urine catecholamines, 5-hydroxyindoleacetic acid, vasoactive intestinal peptide levels, and celiac testing were performed and found to be normal.

A ROHHAD diagnosis was ultimately considered given the rapid weight gain, central hypoventilation, and autonomic dysfunction. Pituitary screening revealed persistently elevated prolactin (50-59 ug/L or 1064-1255 mIU/L; normal range: < 24 ug/L or < 510 mIU/L), with normal macroprolactin. ROHHAD diagnosis was confirmed following the discovery of a 2.9 × 1 × 3 cm left para-adrenal neuroganglioma; although successful laparoscopic resection did not appreciably improve her symptoms.

### Microbiome Assessment

Stool samples were collected during the clinic visit, or at home 1 day prior, kept frozen, transported to the hospital on ice, and stored at −80 °C. Stool genomic DNA was extracted with the QIAamp Fast DNA Stool Mini Kit (QIAGEN) following manufacturer's instructions with bead beating step to enhance dissociation. gDNA concentration was assessed by a NanoDrop 2000C spectrophotometer (Thermo Fisher Scientific).

Amplicon sequencing data for *16S rRNA* variable regions 3 to 4 were generated using previously published primers (2). Library preparation and sequencing were performed at the University of Colorado (Aurora, Colorado). Polymerase chain reaction products were normalized using a SequalPrep^TM^ kit (Invitrogen), pooled, concentrated using a DNA Clean and Concentrator Kit (Zymo), and quantified (Qubit Fluorometer 2.0 Invitrogen). Paired-end sequencing was performed on the Illumina MiSeq platform (Miseq Control Software and MiSeq Reporter v2.4).

An R script (v4.2.2) was written to trim raw reads. Demultiplexed paired-end reads were clustered into amplicon sequencing variants (ASV) using the DADA2 pipeline v1.16 (3). Taxonomic classification was assigned using the Silva *16S rRNA* reference database (v138.1). Diversity analyses were generated using Phyloseq (v3.16), ggplot2 (v3.4.0), and ggalluvial (v0.12.5).

Stool aliquots (0.1-0.2 g) were diluted to 0.2 g/mL in staining buffer (sterile phosphate-buffered saline, 1 mM EDTA, 0.05% Tween-20). Samples were homogenized, centrifuged at 600*g* for 1 minute and the supernatant collected. Then 100 uL of the supernatant was centrifuged at 6000*g* for 10 minutes to recover bacterial cells. Cell suspensions were diluted and stained with Thiazole Orange (Anaspec Inc) and Propidium Iodide (Sigma-Aldrich) for 15 minutes at room temperature. A known concentration of counting beads (Spherotech Inc) was added to stained bacterial cells, and nucleic acid-positive cells were counted on an LSR Fortessa flow cytometer (BD Biosciences, SickKids Flow cytometry facility).

Stool water content was determined from aliquots of 0.1 to 0.2 g as the ratio of mass loss (wet weight/dry weight) after air drying. The wet/dry weight ratio was used to normalize flow cytometry bacterial cell counts in 1 g of dry stool to remove variation imposed from water content differences across stool samples. Absolute abundances were calculated by multiplying the relative abundance of each ASV by normalized bacterial biomass.

## Treatment

Insulin was required for management of type 2 diabetes, reaching a maximal dose of 220 units per day. Treatment with fenofibrate for hypertriglyceridemia was poorly tolerated and was followed by rosuvastatin. On more than one occasion, the patient required admission for fasting, intravenous hydration, and insulin infusion due to extreme hypertriglyceridemia. Weight gain continued, peaking at 104.4 kg (> 99.9%) with a BMI of 41.8 kg/m^2^ (> 99.9%; BMIz +3.42) aged 14 years, despite lifestyle behavior intervention. At age 15, a specialized low carbohydrate diet, protein-sparing modified fast, implemented under close supervision was successful in stabilizing weight gain and improving metabolic status. Ultimately, due to significant metabolic comorbidities, the patient underwent Roux-en-Y gastric bypass (RYGB) surgery at age 17 years.

## Outcome and Follow-Up

**Figure 1.**
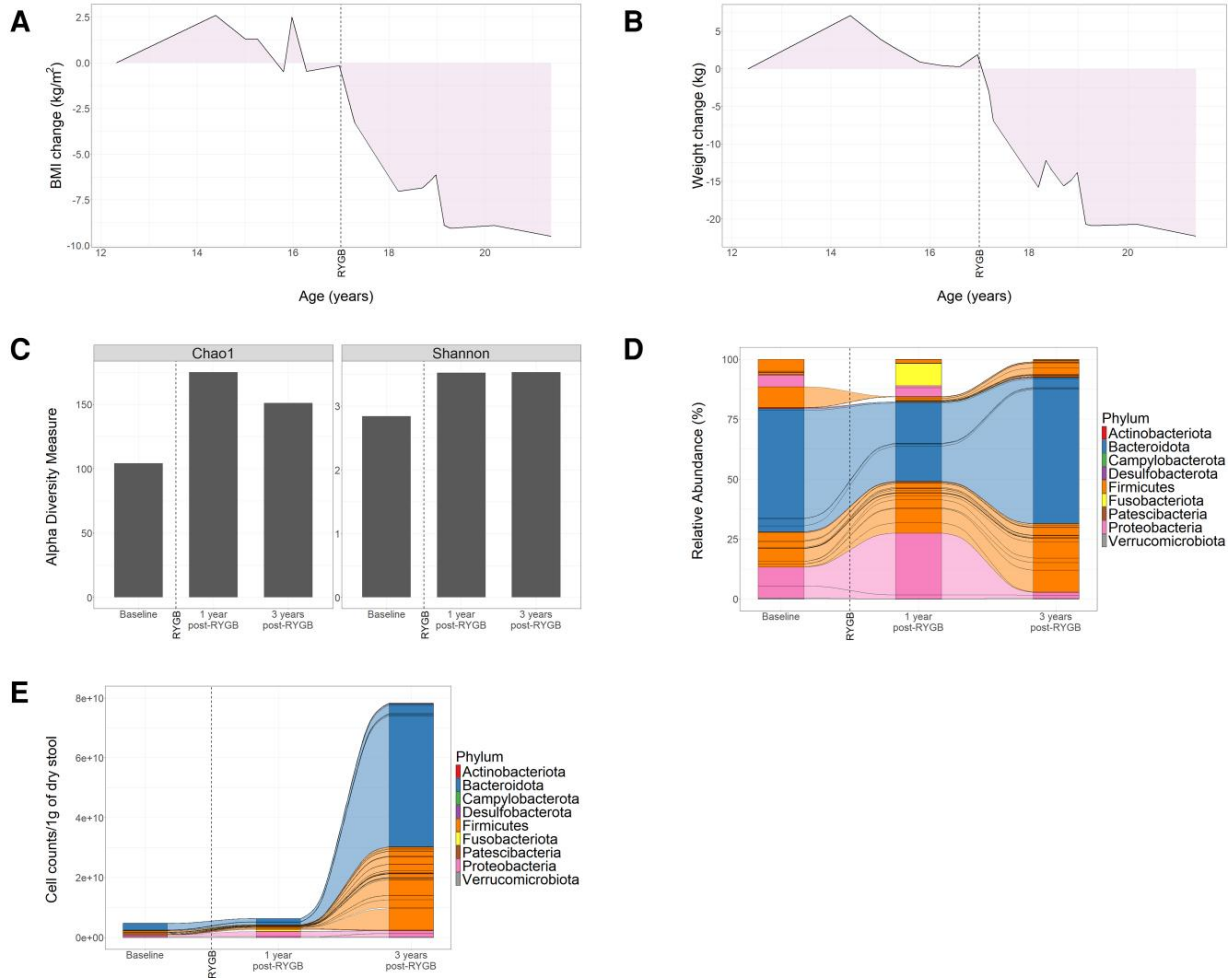
Changes in weight loss and gut microbiome composition and abundance following bariatric surgery in ROHHAD case. Change in BMI (A) and weight (B) from baseline over the course of treatment and follow-up from age 12 to 21 years. The date of Roux-en-Y gastric bypass is annotated by a broken vertical line. Relative abundances of ASVs that represent bacterial composition were identified by *16S* rRNA gene sequencing from stool samples collected at baseline and after surgery (1 year and 3 years post-Roux-en-Y gastric bypass). (C) Bar plots show alpha-diversity measured by Chao-1 and Shannon indices. (D-E) Alluvial plots show relative abundances (D) or absolute abundances (E) of bacterial genera. Lines connecting the columns show identical bacterial genera present across adjacent stool samples. (D) Bacterial genera are grouped by phyla as indicated by color. (E) Stool bacterial biomass was quantified by flow cytometry and normalized by 1 g of dry stool. Alluvial plots show the absolute abundances of each bacterial genus. Bacterial genera are grouped by phyla as indicated by color. Abbreviations: ASV, amplicon sequence variant; BMI, body mass index; RYGB, Roux-en-Y gastric bypass.

Following surgery, the patient lost 22.5 kg over a 24-month period (Fig. 1A and 1B), progressing to a stable weight of 75 kg (BMI 29.7 kg/m^2^). The patient’s metabolic status improved, with weaning of insulin and improvement in HbA1c (6.2%-7.4% or 44-57 mmol/mol), normalization of lipid profile, hepatic enzymes, and resumption of menses. The chronic diarrhea completely resolved shortly after surgery and prior to significant weight loss. Both her central and obstructive sleep apnea resolved allowing discontinuation of positive airway pressure support. Prolactin remained mildly elevated.

Modulation of the gut microbiome following RYGB surgery is likely a key contributor to the improved metabolism after surgery (4). We evaluated bacterial taxonomic composition, diversity, and biomass by *16S rRNA* V3-V4 gene region sequencing in stool samples obtained at baseline (2 years before RYGB) and at 1 and 3 years postoperatively. Bacterial diversity increased after surgery as indicated by both Shannon and Chao-1 indices (Fig. 1C). This was accompanied by changes in the relative abundance of key microbiome phyla. At baseline, Bacteroidota were the most abundant phylum (51.28%), followed by Firmicutes (29.80%) and Proteobacteria (18%; Fig. 1D, Table 1). Transient increases in relative abundances of Fusobacteria (0% to 9.26%) and Proteobacteria (18% to 32%) were observed 1 year after RYGB, followed by contraction 3 years postoperatively (Fig. 1D, Table 1). At 3 years after surgery, Bacteroidota and Firmicutes were the most abundant phyla, accounting for 60.96% and 35.76%, respectively (Fig. 1D, Table 1).

**Table 1.** Changes in gut microbiome composition following bariatric surgery

	Relative abundance (%)		
Phylum	Baseline	1 year post-RYGB	3 years post-RYGB
Actinobacteriota	0.43	0.09	0.35
Bacteroidota	51.28	33.16	60.96
Campylobacterota	0.00	0.06	0.00
Desulfobacterota	0.19	0.13	0.08
Firmicutes	29.80	25.25	35.76
Fusobacteriota	0.00	9.26	0.02
Patescibacteria	0.00	0.00	0.00
Proteobacteria	18.00	32.00	2.57
Verrucomicrobiota	0.30	0.05	0.25

The relative abundances (in percent) of bacterial phyla pre and post Roux-en-Y gastric bypass from Fig. 1D are tabulated.

*Bacteroides* and *Prevotella* were highly abundant genera both at baseline and after surgery. Notably, *Bacteroides* constituted 45% of the stool bacteria at baseline, and contracted to 16.7% and 3.5% at one-year and three-years post-RYGB, respectively. Conversely, *Prevotella* comprised 2.3% of the baseline composition and expanded to 14.7% and 56% at postoperative assessments at 1 year and 3 years, respectively. Several bacterial taxa present at baseline, including members of the genera *Clostridium*, *Morganella,* and *Ruminococcus*, decreased after surgery. In contrast, members of the genus *Acidaminococcus* and *Catenibacterium* increased after surgery, while others were transiently present at year 1 but not year 3 after surgery, including the genus *Fusobacterium* and *Haemophilus*.

Recent studies have associated changes in microbial biomass and stool water content with inflammatory diseases (5-7). This ROHHAD patient had severe diarrhea before surgery, with high stool water content (9.84 g of wet weight/gram dry stool). Consistent with clinical observations, stool water content decreased after RYGB, to 7.87 and 3.1 of wet/dry weight ratio 1 and 3 years postoperatively, respectively. Next, we quantified bacterial cell mass by flow cytometry, normalized to dry stool mass to remove the variability in water content over time. After RYGB, absolute bacterial abundances increased 2.85- and 32.39-fold biomass at 1 and 3 years postoperatively, respectively (Fig. 1E). Bacteroidota and Firmicutes were the main phyla associated with biomass expansion (Fig. 1E).

## Discussion

This case illustrates the challenges of ROHHAD treatment, and the efficacy of bariatric surgery to improve diarrhea, BMI, and cardiometabolic status, all of which were associated with remodeling of intestinal microbial composition and increased biomass. RYGB surgery alters circulating hormones (8), features that likely underpin resulting improvements in weight and glucose-handling (9). The gut microbiome may be a key effector of these hormonal alterations. A systematic review of gut microbiome composition after bariatric surgery uncovered consistent increases in relative abundances of Bacteroidota, Fusobacteria, Verrucomicrobiota, and Proteobacteria (10). Here we report changes in both absolute and relative abundance of these phyla.

The literature conflicts on the link between Bacteroidota abundance and obesity (11, 12). In this case, Proteobacteria and Fusobacteria were increased transiently at 1 year, a phenomenon previously attributed to the rise in intralumenal pH and dissolved oxygen which occurs following such surgeries (13). However, these taxa regressed by the third year of follow-up, suggesting ongoing postoperative gut microbiome remodeling. Similarly, Fusobacteria absence at the final follow-up suggests a shift toward a nonobese microbiome (14). Our observations were advanced by converting microbiome composition from relative to absolute abundances or each taxa. This approach revealed a robust increase in bacterial biomass following RYGB and a concomitant decrease in stool water content. The absolute increases in 2 keystone phyla of healthy human gut microbiota, Bacteroidota and Firmicutes, underpinned this biomass change. Our results suggest that measuring absolute abundance improves longitudinal analysis of microbiome changes associated with disease progression and treatment.

The novelty of this ROHHAD case analysis needs to be tempered by the inherently individual nature of case reporting. Our findings that RYGB produces changes in gut microbiome composition and biomass in ROHHAD, which likely contribute to the resolution of diarrhea and weight loss may motivate research into the gut microbiome in ROHHAD-related obesity and establish RYGB as an effective therapeutic option.

## Learning Points

- ROHHAD syndrome is a rare, poorly understood disorder associated with multimorbidity and a high risk of mortality.
- This case outlines the first successful correction of obesity, dysautonomia, and metabolic derangement in one ROHHAD patient following Roux-en-Y gastric bypass.
- Bariatric surgery resulted in sustained changes in gut microbiome composition and biomass in this case.

## Data Availability

Restrictions apply to the availability of some or all data generated or analyzed during this study to preserve patient confidentiality or because they were used under license. The corresponding author will on request detail the restrictions and any conditions under which access to some data may be provided.

## Abbreviations

ASV: amplicon sequence variant
BMI: body mass index
HbA1c: glycated hemoglobin
ROHHAD: rapid onset obesity with hypoventilation, hypothalamic, and autonomic dysregulation
RYGB: Roux-en-Y gastric bypass
